# Maternal Health, Neonatology, and Perinatology reviewer acknowledgement 2015

**DOI:** 10.1186/s40748-016-0029-6

**Published:** 2016-02-18

**Authors:** Tonse Nk Raju

**Affiliations:** National Institutes of Health, 31 Center Drive, Bethesda, MD 20892-2425 USA

## Abstract

The editor of *Maternal Health, Neonatology, and Perinatology* would like to thank all of the reviewers who have contributed to the journal in Volume 1 (2015).

**Alexandria Hill**

United States of America

**Alireza Shamshiraz**

United States of America

**Anup Katheria**

United States of America

**Ashley Williams**

United States of America

**Carlos Blanco**

Ireland

**Christina Herrera**

United States of America

**David Hutchon**

United Kingdom

**Devaraj Sambalingam**

United States of America

**Don McCurnin**

United States of America

**Elizabeth McClure**

United States of America

**Heike Rabe**

United Kingdom

**Jayashree Ramasethu**

United States of America

**Jonathan Samet**

United States of America

**Jorge Tolosa**

United States of America

**Kelli Barbour**

United States of America

**Malissa Parisi**

United States of America

**Martin Keszler**

United States of America

**Matthew Laughon**

United States of America

**Nithi Fernandes**

United States of America

**Payam Vali**

United States of America

**Praveen Chandrasekharan**

United States of America

**Robert Silver**

United States of America

**Rodriguez Christina**

United States of America

**Roland Hentschel**

Germany

**Rosemary Higgins**

United States of America

**Ruth Grunau**

Canada

**Satyan Lakshminrusimha**

United States of America

**Stuart Hooper**

Australia

**Vikramaditya Dumpa**

United States of America

**Vinay Sharma**

United States of America

**Ware Branch**

United States of America

**William Zorn**

United States of America

**Yawei Zhang**

United States of America

